# Supplementary material for: The impact of COVID-19 workload on psychological distress amongst Canadian intensive care unit healthcare workers during the 1st wave of the COVID-19 pandemic: A longitudinal cohort study
Source: PLoS One. 2024 Mar 7;19(3):e0290749. doi: 10.1371/journal.pone.0290749 (PMC10919682; doi:10.1371/journal.pone.0290749)
Supplement: S1 Appendix — (PDF) [file pone.0290749.s001.pdf]

COVID-HCW Survey Advertisement

COVID-19 Exposure and Transmission Amongst Canadian ICU Healthcare Workers (COVID-HCW Survey)

The **COVID-HCW Survey** was designed by a team of frontline healthcare workers (HCW) to collect information regarding:

- HCWs' exposure to COVID-19 patients
- COVID-19 transmission to HCWs
- HCWs' use of personal protective equipment (PPE)
- HCWs' comfort surrounding the use of PPE
- HCWs' overall psychosocial wellbeing

Our goal is to understand the COVID-19 exposure and transmission risks amongst Canadian ICU healthcare workers longitudinally during the COVID-19 pandemic.

We are seeking responses from all frontline healthcare workers (physicians, nurses, respiratory therapists, physiotherapists, occupational therapists, pharmacists, dietitians, etc) directly involved in the clinical care of ICU patients with suspected and/or confirmed COVID-19 through this weekly longitudinal survey on exposure/transmission risks and psychosocial stress level.

If you are interested, please click:

COVID-HCW Study Consent and Enrolment Link

The COVID-HCW Survey Project has been reviewed by the Hamilton Integrated Research Ethics Board.

Thank you for your consideration to participate in this longitudinal survey.

COVID-HCW Survey Team

Jennifer Tsang, MD, PhD, FRCPC
Intensivist, Niagara Health
Alexandra Binnie, MD, DPhil, FRCPC
Intensivist, William Osler Health System
