# Supplementary material for: The impact of COVID-19 workload on psychological distress amongst Canadian intensive care unit healthcare workers during the 1st wave of the COVID-19 pandemic: A longitudinal cohort study
Source: PLoS One. 2024 Mar 7;19(3):e0290749. doi: 10.1371/journal.pone.0290749 (PMC10919682; doi:10.1371/journal.pone.0290749)
Supplement: Attachment — Submitted filename: Response to reviewers.docx [file pone.0290749.s004.docx]

**Response to reviewers:**

Dear PLOS ONE Editorial Team,

Thank you for taking the time to review our manuscript (PONE-D-23-09335). Please see our detailed response to reviewers below.

Yours sincerely,

Dr Jennifer Tsang

**Journal requirements:**
When submitting your revision, we need you to address these additional requirements.

1. Please ensure that your manuscript meets PLOS ONE's style requirements, including those for file naming. The PLOS ONE style templates can be found at

https://journals.plos.org/plosone/s/file?id=wjVg/PLOSOne_formatting_sample_main_body.pdf and

https://journals.plos.org/plosone/s/file?id=ba62/PLOSOne_formatting_sample_title_authors_affiliations.pdf

2. Please provide additional details regarding participant consent. In the ethics statement in the Methods and online submission information, please ensure that you have specified what type you obtained (for instance, written or verbal, and if verbal, how it was documented and witnessed). If your study included minors, state whether you obtained consent from parents or guardians. If the need for consent was waived by the ethics committee, please include this information.

We have added the following statement to the methods section: “*Electronic consent was provided by participants prior to completion of the T1 survey. Withdrawal was allowed at any time and participants could also request removal of their data. Participants who did not reply to two consecutive surveys were sent an email asking if they wished to continue in the study. During the 16 weeks of the study, 64 participants withdrew from further participation and 1 participant asked for their data to be removed*.”

3. We note that the grant information you provided in the ‘Funding Information’ and ‘Financial Disclosure’ sections do not match.

When you resubmit, please ensure that you provide the correct grant numbers for the awards you received for your study in the ‘Funding Information’ section.

Funding for this study was provided by Mohawk Medbuy Corporation, which is a non-profit, shared services corporation. Mohawk Medbuy does not have a formal granting program and there is no grant number associated with the funding. The money was provided to JT as an unrestricted research grant in April 2020 and the funder had no role in study design, data collection and analysis, decision to publish, or preparation of the manuscript.

4. Thank you for stating the following in the Funding Section of your manuscript:

“This study was funded by an unrestricted research grant to JT from the non-profit Mohawk-Medbuy Corporation (https://www.mohawkmedbuy.ca/). The funders had no role in study design, data collection and analysis, decision to publish, or preparation of the manuscript. “

We note that you have provided funding information that is not currently declared in your Funding Statement. However, funding information should not appear in the Acknowledgments section or other areas of your manuscript. We will only publish funding information present in the Funding Statement section of the online submission form.

The funding information appears only in the Funding Statement section of the manuscript.

Please remove any funding-related text from the manuscript and let us know how you would like to update your Funding Statement. Currently, your Funding Statement reads as follows:

“The funders had no role in study design, data collection and analysis, decision to publish, or preparation of the manuscript.” Please include your amended statements within your cover letter; we will change the online submission form on your behalf.

The Funding Statement should be identical to what is written in the funding section of the manuscript, which is:

This study was funded by an unrestricted research grant to JT from Mohawk Medbuy, a not-for-profit, shared services corporation (<https://www.mohawkmedbuy.ca/>). The funder had no role in study design, data collection and analysis, decision to publish, or preparation of the manuscript.

5. In your Data Availability statement, you have not specified where the minimal data set underlying the results described in your manuscript can be found. PLOS defines a study's minimal data set as the underlying data used to reach the conclusions drawn in the manuscript and any additional data required to replicate the reported study findings in their entirety. All PLOS journals require that the minimal data set be made fully available. For more information about our data policy, please see http://journals.plos.org/plosone/s/data-availability.

Upon re-submitting your revised manuscript, please upload your study’s minimal underlying data set as either Supporting Information files or to a stable, public repository and include the relevant URLs, DOIs, or accession numbers within your revised cover letter. For a list of acceptable repositories, please see http://journals.plos.org/plosone/s/data-availability#loc-recommended-repositories. Any potentially identifying patient information must be fully anonymized.

Important: If there are ethical or legal restrictions to sharing your data publicly, please explain these restrictions in detail. Please see our guidelines for more information on what we consider unacceptable restrictions to publicly sharing data: http://journals.plos.org/plosone/s/data- availability#loc-unacceptable-data-access-restrictions. Note that it is not acceptable for the authors to be the sole named individuals responsible for ensuring data access.

We will update your Data Availability statement to reflect the information you provide in your cover letter.

We have included the minimal underlying dataset with our submission and have included the data statement in our cover letter.

6. Please review your reference list to ensure that it is complete and correct. If you have cited papers that have been retracted, please include the rationale for doing so in the manuscript text, or remove these references and replace them with relevant current references. Any changes to the reference list should be mentioned in the rebuttal letter that accompanies your revised manuscript. If you need to cite a retracted article, indicate the article’s retracted status in the References list and also include a citation and full reference for the retraction notice.

Additional Editor Comments:

Please revise the manuscript according to the reviewers' comments and upload the revised file. Any revisions should be “clearly highlighted”, for example using the Track Changes function in Microsoft Word or using a different colour, so that they are easily visible to the editors and reviewers.
Please provide a short Cover letter detailing any changes, for the benefit of the editors and reviewers.

**Comments to the Author**

1. Is the manuscript technically sound, and do the data support the conclusions?

The manuscript must describe a technically sound piece of scientific research with data that supports the conclusions. Experiments must have been conducted rigorously, with appropriate controls, replication, and sample sizes. The conclusions must be drawn appropriately based on the data presented.

Reviewer #1: Yes Reviewer #2: Yes

2. Has the statistical analysis been performed appropriately and rigorously? Reviewer #1: Yes
Reviewer #2: Yes

3. Have the authors made all data underlying the findings in their manuscript fully available?

The PLOS Data policy requires authors to make all data underlying the findings described in their manuscript fully available without restriction, with rare exception (please refer to the Data Availability Statement in the manuscript PDF file). The data should be provided as part of the manuscript or its supporting information, or deposited to a public repository. For example, in addition to summary statistics, the data points behind means, medians and variance measures should be available. If there are restrictions on publicly sharing data—e.g. participant privacy or use of data from a third party—those must be specified.

Reviewer #1: Yes Reviewer #2: Yes

4. Is the manuscript presented in an intelligible fashion and written in standard English?

PLOS ONE does not copyedit accepted manuscripts, so the language in submitted articles must be clear, correct, and unambiguous. Any typographical or grammatical errors should be corrected at revision, so please note any specific errors here.

Reviewer #1: Yes Reviewer #2: Yes

5. Review Comments to the Author

Please use the space provided to explain your answers to the questions above. You may also include additional comments for the author, including concerns about dual publication, research ethics, or publication ethics. (Please upload your review as an attachment if it exceeds 20,000 characters)

Reviewer #1: In this paper, the authors present a longitudinal study of mental health among Canadian healthcare workers (HCWs) from intensive care units (ICU). The population was studied longitudinally at the beginning, middle and end of the first wave of Covid in 2020. A total 309 HCWs participate in the first survey timepoint, and around 190 at each of the two subsequent rounds of survey. Overall, the authors identified several factors that contributed to the presence of psychological distress at the various timepoints, as well as identifying factors associated with the likelihood of recovery from the distress.

The authors are to be commended for mounting such a comprehensive project at such short notice. The results are interesting, and the paper is generally well written.

I have some comments for the authors’ consideration:

Methods : page 4, lines 109-111 : were the data about workload self-reported? Or were they retried from written hospital administrative data? If self-reported, this may be subject to bias and should be mentioned as a limitation.

Thank you for this comment. Workload data were self-reported but were collected weekly to minimize recall bias. We have added this as a limitation of the study.

Methods / survey administration: I appreciate that the authors provided additional methodological information in the supplementary material, but to facilitate the reading of the main paper, more detail is needed here, on two key points:
First, the authors should explain exactly how the survey was distributed – was it simply emailed to all registered members of the societies listed? Do the authors have any idea of the number of people who received the link? Could recipients forward the link to other people?

Thank you for this question. Distribution of the survey link included social media platforms associated with Canadian critical care professional associations so the number of recipients is unknown. Moreover, anyone with access to the link could enrol. It is therefore impossible to calculate a response rate. We have clarified this in the methods section.

Second, it is unclear what exactly the participants were required to do. From reading the results on page 6, paragraph “Covid 19 workload”, and from my reading of the consent form provided in the supplementary material, it appears that the participants were asked to provide information about workload on a weekly basis. This is not clear from the methods, where the reader is given to understand that the participants were surveyed at 3 timepoints only (and not in between). The authors should clearly explain what was measured at the 3 timepoints T1, T2 and T3, and specify whether the participants also had to provide information about their workload every week. If so, please explain how this was done. It’s not explained in the supplementary methods either. I cannot understand fully, from the methods presented, whether the participants were individually identified and followed – i.e. are the 195 and 191 respondents who participated in the surveys at T2 and T3 respectively, a subset of the original 309 participants from T1? If they were all recording their workload weekly, that indicates a high drop-out rate, with about one third of the participants lost by T2.

Thank you for pointing out this issue with the methods. We have now clarified how the study was structured. In brief, after completing the e-consent and the T1 survey, participants completed weekly surveys for 16 weeks about their COVID-19 workload as well as longer surveys at the T2 (week 9) and T3 (week 16) timepoints. Weekly responses were consistently in the range of 150 - 220 participants. The mean number of responses per participant was 9.23 (SD = 5.22). The number of participants who completed only the enrolment survey was 24.

Results, page 8, line 231/232: if weekly hospital shifts is associated with a protective effect, then it should be specified in Table 3 that the OR is the increase in risk per additional shift worked. Same remark for weeks shifts with Covid exposure.

We have added this information to the table legend.

Results, page 10, line 248 – do the authors mean that there are 190 unique individuals who were identified to have answered both surveys?

This is correct. We have clarified this in the text.

Table 4 – I don’t recall it being mentioned in the methods that the BRCS was administered – when was this tool administered to participants? This should be added to the methods section.

We have amended the methods section to include this information. It was administered at the T3 timepoint.

Table 5 – I think the title of this table is wrong, or else the authors have interpreted the data the wrong way around (unlikely). On page 11, lines 272-274, the authors report the factors associated with the likelihood of recovery from psychological distress, and these factors are what Table 5 purports to report. However, the authors report that male sex and a higher number of Covid patients in the ICU were associated with recovery – but this is ambiguous. According to the odds ratios reported in Table 5, both these factors have ORs that are associated with a protective effect, and therefore, a lower likelihood of the outcome. If the outcome modelled in the regression analysis is “recovery”, then male sex and more COVID cases in ICU “protect against” recovery and thus, are associated with a lower likelihood of recovery. This is incongruent with the result report on lines 273/274, where the authors state that physician profession and a higher number of weekly shifts with exposure to Covid patients were associated with persistence of psychological distress. Firstly, there is no odds ratio for the profession “physician”, indicating that this category was the reference category, so it cannot be associated with the outcome. There must a mistake somewhere. Secondly, the odds ratio associated with the number of hospital shifts with exposure is 1.97, indicating an approximately twofold increase in the risk of the outcome. This suggests that the outcome being modelled in the table is “persistent psychological distress” (i.e. lack of recovery), which is the opposite of what the table title indicates. So, overall, there is some work to be done here to ensure that the table title clearly indicates the outcome being modelled, to enable the reader to interpret the ORs in the right direction. Second, the authors must clarify the reference category used among the professions, and correct the result pertaining to physicians. The same comment applies to the discussion, because the authors discuss this result about physician recovery (page 12, line 335), but the reader does not see this result in the results section.

Thank you for identifying this error. We have corrected both the table title and the discussion to correctly report the results of the regression.

A key limitation of this study is that there is no information about baseline mental health among the respondents. Indeed, it has been reported that pre-pandemic, around 13% of physicians/surgeons and around 18% of registered nurses may have had diagnosed depression (DOI: 10.1097/JOM.0000000000002630). Therefore, we do not know how many of these professionals had mental health issues or signs and symptoms of burnout before the pandemic began.

This is a very relevant issue and we have included this in the discussion.

Discussion, page 12, line 323: the authors state that “these participants appear to have been more susceptible to mental health issues at T3” – what is your basis for this assertion?

Thank you for this comment. We have corrected the text to indicate that there was a higher prevalence of psychological distress in this subgroup of participants rather than greater susceptibility.

Limitations – as mentioned above, the authors might add the lack of knowledge about baseline mental health status, and possible bias in the reporting of workload. Also, since we are now more than three years later, the authors might mention how this information can be of use to the medical community now.

We have added baseline mental health status and reporting bias as limitations of the study. We have also added discussion relating to how this study can influence future pandemic preparedness.

References: the authors should verify the format of the references – some still contain dates (e.g.refs 5,6, 7....), and some journal names are not presented correctly (e.g. ref 10...)

Thank you for pointing out this formatting error. We have verified the references to ensure that the style is correct.

In summary: The main action points include a revision of the methods to make the exact procedures clearer; clarify the interpretation of Table 5 and the related text in the results and discussion; and add limitations as detailed above.

Reviewer #2: Thank you for the opportunity to review this manuscript. I commend the authors on conducting this important study and at such an early and chaotic phase of the pandemic. It is impressive that they had a relatively high follow-up rate as part of this longitudinal survey, which is notoriously challenging to achieve, especially in studies of healthcare providers.

I have included suggestions below for the authors to consider.

Abstract
- The abstract does not currently include statistical data from the multivariable analysis, although the findings are described in writing. Generally, the data are included for readers to take the strengths of any associations into consideration. Would suggest the authors include the few relevant data points numerically for findings that they have summarized in writing.

Thank you for this suggestion. We have amended the abstract to include the data points.

Introduction
- The Introduction is well-written and referenced well. Given that this is a Canadian study with a large proportion of participants from Ontario, perhaps the authors can consider summarizing other Canadian/ Ontario studies that have been published in the literature... numerous have been published that can be incorporated... a few of these listed below:
> PMID: 34940952
> PMID: 35167590
> PMID: 36627462

Thank you for this suggestion. We have added Canadian data to the introduction.

- The last paragraph of the Introduction section begins with "Only a few studies..." but these studies have not been cited. Please cite the studies you're referring to.

Thank you for pointing out this omission. We have cited the relevant studies in the introduction. They are also discussed in the discussion section.

- The objective statement (last 2 sentences in the introduction) are detailed enough that they are similar to statements in the Methods. I would suggest a broad objective statement be included instead to summarize the overall purpose of the study, without getting into Methodology at this stage.

Thank you for this suggestion. We have amended the final paragraph of the introduction so that it is less methodological and provides an overview of the objective of the study.

Materials and Methods

- The study is well described in this section.

Results
- What proportion of participants were from academic vs. community settings? This is important to note given my comment below regarding the authors' speculation about community ICU physicians.

Unfortunately, this information is not available to us as participants were not required to provide their affiliation. We have corrected the discussion to remove the reference to “community” ICU physicians.

- The authors describe the findings of the BRCS in Table 4 but did not indicate in the Methods that this instrument was also administered and do not discuss its findings in the Results. This is an interesting variable and would be of interest to readers to know about the outcomes on the resilience front. I would suggest discussing this instrument in the Methods and Results in more detail.
- Also regarding the BRCS, was this variable considered for inclusion in the MV analysis?

Thank you for pointing out this omission. We have added the information on the BRCS administration to the methods section. The BRCS was included as a potential variable in the MV analysis.

- Table 3 - I would suggest putting the most notable findings where a variable was predictive of the outcome in bold font for easier review by readers.

Thank you for this suggestion. The final MV model was determined using stepwise forward and back regression to obtain the lowest AIC. As such, all variables belong in the final model. We therefore did not feel that “significant” p-values should be highlighted. We did highlight the significant p-values in other analyses.

- Table 5 indicates that the physician profession was used as the reference variable for the analysis but the text states that the physician profession was predictive of persistence - can the authors please explain and provide the data that led to this conclusion? Was there another analysis in which the physician profession was not used as the reference variable and allowed the authors to draw this conclusion?

Thank you for pointing out this error of interpretation. Physicians showed greater persistence of psychological distress than RTs and allied health professionals, but not RNs. We have clarified this in the discussion section.

- The authors state that physician role was predictive of persistence of mental health issues - was the nursing profession (which showed the highest proportion of respondence with persistence) not predictive of persistence? If not, how do the authors explain this finding? Is it possible that this finding could be explained by multicollinearity between sex and the nursing profession (since most nurses were female)? I imagine that would influence the MV analysis and perhaps, it may not be appropriate to include both nursing profession and sex in the same model due to high collinearity issues?

Thank you for this suggestion. We have checked for multicollinearity in the dataset using the variance inflation factor and there was no significant multicollinearity. As you rightly pointed out, no significant difference was identified between nurses and physicians with respect to persistence of psychological distress. We have clarified this in the discussion section.

Discussion
- As noted in my comment about the Introduction, it would be great if the authors could discuss their findings in the context of other Canadian- based studies.

We have added Canadian data to the introduction section. Unfortunately, the longitudinal studies that are most comparable to our study are not from Canada and we were unable to find any similar longitudinal Canadian studies to include in the discussion.

- The first paragraph of the Discussion section is a reiteration of the Intro and Methods - I would propose this is not necessary and the Discussion should usually begin with a high-level summary of the study findings.

Thank you for this suggestion. We have amended the discussion to provide a high-level summary of findings.

- The authors discuss the isolated work environment of community ICU physicians but did not state whether this variable specifically (being a community ICU physician) was an independent predictor of poorer mental health. Perhaps the MD group has a small sample size and would not allow for such an analysis. At a minimum, would suggest reporting the number of community vs. academic physicians in the sample.

Unfortunately, participants were not asked to provide a hospital affiliation so we don’t know how many were working in community vs academic ICUs. However, the study was publicized through the Canadian Community ICU Research Network and we expect that some of the physician participants were working in community ICUs. Moreover, the study leads (Binnie and Tsang) are both community ICU physicians, which may have prompted more community ICU physicians to enrol. Given the uncertainty around practice location we have removed the term “community ICU physician” from the discussion section but have maintained mention of the potential impact of working in isolation on physician mental health.

Conclusion
- Here, the authors discuss 'external factors, such as news media coverage...' which they don't explicitly describe in the Discussion section as potential contributors to poorer mental health. I would suggest the authors discuss this in more detail in the discussion, so that they are not discussing a new concept in the Conclusion section.

Thank you for this comment. At the beginning of the discussion section we refer to the proliferation of news coverage about COVID-19 at the start of the pandemic and how this may have impacted mental health of HCW, as well as the general public.

6. PLOS authors have the option to publish the peer review history of their article (what does this mean?). If published, this will include your full peer review and any attached files.

If you choose “no”, your identity will remain anonymous but your review may still be made public.
**Do you want your identity to be public for this peer review?** For information about this choice, including consent withdrawal, please see

our Privacy Policy.
Reviewer #1: **Yes:** Fiona Ecarnot Reviewer #2: No

[NOTE: If reviewer comments were submitted as an attachment file, they will be attached to this email and accessible via the submission site. Please log into your account, locate the manuscript record, and check for the action link "View Attachments". If this link does not appear, there are no attachment files.]

While revising your submission, please upload your figure files to the Preflight Analysis and Conversion Engine (PACE) digital diagnostic tool, https://pacev2.apexcovantage.com/. PACE helps ensure that figures meet PLOS requirements. To use PACE, you must first register as a user. Registration is free. Then, login and navigate to the UPLOAD tab, where you will find detailed instructions on how to use the tool. If you encounter any issues or have any questions when using PACE, please email PLOS at figures@plos.org. Please note that Supporting Information files do not need this step.

*In compliance with data protection regulations, you may request that we remove your personal registration details at any time. (Remove my information/details). Please contact the publication office if you have any questions.*
